# Electrophysiologic evidence of loss of consciousness in cattle during slaughter with and without stunning: a systematic review and methodological overview

**DOI:** 10.3389/fvets.2026.1809389

**Published:** 2026-05-18

**Authors:** Sheryl R. Haut, Thomas Parmentier, Robert S. Fisher, Nofal M. Khalil

**Affiliations:** 1 Department of Neurology, Montefiore-Einstein, Bronx, NY, United States; 2 Department of Clinical Sciences, Faculty of Veterinary Medicine, Université de Montreal, Montreal, QC, Canada; 3 Department of Neurology and Neurological Sciences, Stanford University School of Medicine, Palo Alto, CA, United States; 4 Department of Clinical Neurophysiology, Imperial College Healthcare NHS Trust, London, United Kingdom

**Keywords:** cattle, consciousness, electrocorticography, electroencephalography, evoked potentials, halal, shechita, slaughter

## Abstract

**Background:**

Slaughter without prior stunning as practiced under Jewish and Islamic religious law raises certain particular animal welfare issues, notably the time between the incision and loss of consciousness (LOC). LOC marks the point at which cortical processing required for perception, including pain, is no longer possible. Quantifying time to LOC in animals is challenging. To date, the most direct and potentially objective means of doing so is the use of electrophysiologic methods. These are the focus of this paper.

**Methods:**

This paper includes a concise discussion of electrophysiology as applied to slaughter, addressing a critical gap in foundational electrophysiologic knowledge required to interpret LOC and insensibility in the veterinary literature. Then, using a PRISMA-guided systematic approach, this review synthesizes neurophysiologic evidence on time to LOC in cattle. A review was conducted using PubMed/MEDLINE, Web of Science, Cochrane Library, and Google Scholar (through December 2025). Experimental cattle studies using EEG, ECoG, or evoked potentials were included, while non-bovine, behavioral-only, or methodologically insufficient studies were excluded. Nine studies were included, and risk of bias was assessed using ROBINS-I.

**Results:**

Overall risk of bias across included studies was judged to be moderate. Studies using electrocorticography (ECoG) reported electrophysiologic markers consistent with LOC occurring between 4.4 and 13 seconds after incision, with mean values ranging from 7.5 to 10.8 seconds. Reported times vary across studies, particularly those using scalp EEG, likely due to methodological differences and limitations.

**Conclusions:**

The highest-quality available electrophysiologic data, suggests that LOC occurs rapidly following slaughter without stunning.

## Introduction

Ensuring a brief interval from ventral neck incision to loss of consciousness (LOC) is a fundamental principle of humane slaughter practices. Neurophysiologic testing has been instrumental in answering key questions about slaughter of cattle for food consumption without prior stunning. Specifically, how much time elapses between the slaughter incision and the animal losing consciousness? Among available approaches, neurophysiologic assessments provide a means of addressing this question, drawing on techniques originally developed for human clinical use and subsequently adapted for veterinary applications. These include electroencephalography (EEG), electrocorticography (ECoG), and evoked potentials (EP), all of which have been used to evaluate both categories of animal slaughter for food, namely conventional slaughter with pre-stunning and slaughter without stunning (sometimes called religious or ritual slaughter) methods. Stunning prior to slaughter is prohibited under Jewish and many interpretations of Islamic religious law which require the animal to be conscious and unblemished at the time of slaughter. The techniques employed in Jewish religious slaughter, called Shechita, and Islamic slaughter, called Dhabiha, differ in their specifics, but both involve a rapid cut that severs the carotid arteries, jugular veins and trachea (1). Conducting neurophysiologic assessments of consciousness during slaughter presents many methodological challenges. These include variations across animal species, method of slaughter, rate of change to cerebral perfusion, and the type of neurophysiologic testing employed. The greatest variation in time to LOC in slaughter without stunning of animals exists in the bovine (cattle) literature (2). In this paper, we review EEG, ECoG, and related EP studies in cattle following slaughter with and without pre-slaughter stunning, grounded on foundational neurophysiological principles. This review addresses one aspect of animal welfare at slaughter, the time to LOC following the slaughter incision. It is also important to acknowledge that concerns regarding animal welfare in slaughter without stunning extend beyond time to LOC. These include the potential for nociceptive activation at the time of incision, variability in cut quality and position and stress associated with handling and restraint prior to slaughter. These factors may influence the animal's overall experience and are frequently cited in the broader welfare literature (3–7). The present review does not attempt to quantify or resolve these considerations. Finally, because the interpretation of electrophysiologic measures is complex, a brief neurophysiologic background is provided which focuses on the interpretive differences between EEG, ECoG, and EPs. Those differences are central to understanding the evidence synthesized in this review.

### Comparing human and bovine brains

The principles governing the proper interpretation of EEG, ECoG and EP results are primarily learned from human studies. However, there are obvious differences between the human and bovine brain. As Dawkins (8) warned about comparing consciousness in humans and animals, “extrapolations from one species to another are not justified without a detailed understanding of the underlying mechanisms” (8). Therefore, this overview must be approached with an eye toward appreciating both the similarities and contrasts in brain structure and function of the human and bovine brains, both in relation to the underlying neuroscience and in the appropriateness of different testing modalities.

### Anatomical and functional differences

The human brain is two times larger and weighs up to three times more than the bovine brain (9). Humans have an extensive prefrontal cortex that supports complex reasoning, abstract thinking and advanced behavioral responses, along with specialized cortical areas for language processing. Bovine brains have a less elaborated prefrontal cortex relative to humans, while possessing highly developed olfactory systems and visual pathways adapted for panoramic rather than binocular vision (10). Despite these differences, there are many brain similarities which support the utilization of data from human neurophysiology for understanding bovine cerebral activity. The brains are similar in terms of gestational development and differentiation, as both humans and bovines are long gestation species with significant brain development occurring by the second trimester. This late development results in relatively large brains with a highly gyrencephalic (convoluted) cortex and brain surface (11). Furthermore, the mature brains of both species exhibit comparable structural and functional organization, with similar brainstem, cerebellar, and cerebral cortical regions performing analogous regulatory functions (9). While the cerebellar and vestibular areas of the bovine brain are developed to support quadruped motor control, the core neural architecture remains comparable to that of humans, showing a basic histological uniformity (12). Both have a six-layer neocortex with similar architecture and neurons in layer VI. These features promote the ability to record reliable and reproducible electroencephalographic data from both human and bovine brains.

### Neurophysiology of slaughter with stunning

A stunned animal undergoes significant damage to the brain and therefore its neurophysiology differs markedly from that of an animal that has been slaughtered without stunning and, therefore, has an intact brain. Electrical stunning may be applied as head-only stunning which induces a reversible epileptiform state requiring subsequent exsanguination to cause death, or as head-to-body stunning, which can induce both immediate unconsciousness and cardiac arrest.

Similar to human seizures occurring in epilepsy, the generalized seizure triggered by successful electrical stunning of an animal causes the animal to become fully unconscious immediately (13, 14). The EEG following a generalized tonic-clonic seizure is characterized by generalized voltage suppression and subsequent slowing of cortical frequencies which lasts from minutes to hours (15).

Captive bolt stunning is a technique in which a stunner delivers a concussive force, either penetrating or non-penetrating, to the skull of an animal to induce LOC prior to exsanguination. Penetrating captive bolt produces direct and extensive disruption of brain tissue along with significant acceleration forces, whereas non-penetrating captive bolt primarily generates rapid acceleration of the head and a consequent acute rise in intracranial pressure. These effects frequently precipitate a generalized tonic seizure, characterized by limb rigidity, often followed by clonic movements (16, 17), although seizure activity is not universal. Overall, captive bolt stunning induces immediate LOC through profound mechanical disruption of brain structures.

### Neurophysiology of slaughter without stunning

Slaughter without prior stunning does not involve the application of electricity or a physical impact to the brain. Instead, LOC is induced by the swift severing of the ventral neck structures and vessels which causes an immediate, catastrophic loss of cerebral blood flow and perfusion pressure to the head/brain of the animal (18–21). Additionally, vertebral blood flow (18, 22, 23) and vertebral artery blood pressure (24–26) decreases to negligible levels, insufficient to sustain cortical perfusion and therefore cortical function (20, 21, 27). Recent review articles conclude that the sudden disruption of cerebral circulation in the context of slaughter without stunning leads to an irreversible and near-immediate loss of cortical function (20, 21, 27). In this context, “immediate” refers to the rapid hemodynamic collapse occurring within 1–3 s following carotid transection (18–21, 24, 28), whereas electrophysiologically detectable LOC occurs typically in the range of approximately 4–13 s in higher-quality studies (29). This rapid reduction in cerebral perfusion provides a strong physiological substrate for the observed cerebral electrophysiological changes following ventral neck incision. Although longer intervals (e.g., 5–336 s) have been reported in some EEG-based studies (14, 30–34), these longer times likely reflect methodological limitations rather than true delays in cortical loss of function (29).

### Neurophysiological techniques

#### Electroencephalography (EEG) overview

EEG measures electrical activity generated from neurons in the cerebral cortex. This activity is captured using electrodes placed in prespecified arrays, and analyzed by comparing the activity recorded in pairs of electrodes. Examining frequencies of the recorded activity helps to classify behavior and function. The background activity in EEG recordings ranges from normal patterns across the full frequency spectrum, to focal or diffuse cerebral dysfunction characterized by increased slow-wave activity (including delta and theta frequencies), and ultimately to total loss of cortical activity (isoelectric recording). The following definitions have been developed from human electroencephalography but are applicable to bovine studies for the reasons discussed above (see Comparing human and bovine brains).

### EEG frequencies

Normal awake EEG: The normal awake EEG consists of desynchronized activity of varying frequencies, typically in the alpha (8–12 Hz) and beta (13 Hz and greater) frequency range. Depending on age and cerebral region, frequencies in the theta (4–8 Hz) range may be normal. For the most part, slow delta frequency waves (<4 Hz) are not normal in the awake brain. Slow wave activity: While low-frequency activity (delta and theta bands) is present to some extent in the normal awake EEG, a predominance of slow wave activity is not typical in the awake state and is most commonly observed during drowsiness or sleep (35). Outside of normal sleep-related transitions, increased or dominant slow wave activity generally reflects cortical dysfunction (36). Theta waves indicate mild dysfunction while delta waves indicate more advanced dysfunction (37). When these slow waves are focal, they indicate that a particular area of the brain is injured (38).

Generalized slowing: This pattern when present in the EEG indicates a global state change. The term HALF, which is utilized in many of the studies examined below, stands for High Amplitude Low Frequency waves. This term describes a pattern of generalized delta wave slowing. While HALF waves may be seen at baseline in a few other unusual scenarios (39–41), their sudden appearance in the context of slaughter is recognized as representing the onset of LOC (2, 29, 42–44). HALF activity can represent a recoverable state of unconsciousness under certain conditions, such as syncope, where cerebral perfusion is transiently reduced within a closed vascular system. In contrast, during slaughter by ventral neck incision, rapid and sustained loss of cerebral blood flow and perfusion pressure due to open-system hemorrhage renders the onset of HALF activity indicative of irreversible cortical failure and, hence, LOC. Isoelectric EEG: A flat line or isoelectric EEG (also termed electrocerebral silence) is consistent with complete loss of cortical cerebral activity and therefore function, including brain death. In fact, EEG is sometimes utilized in the determination and confirmation of brain death.

### EEG techniques

**Figure 1:**
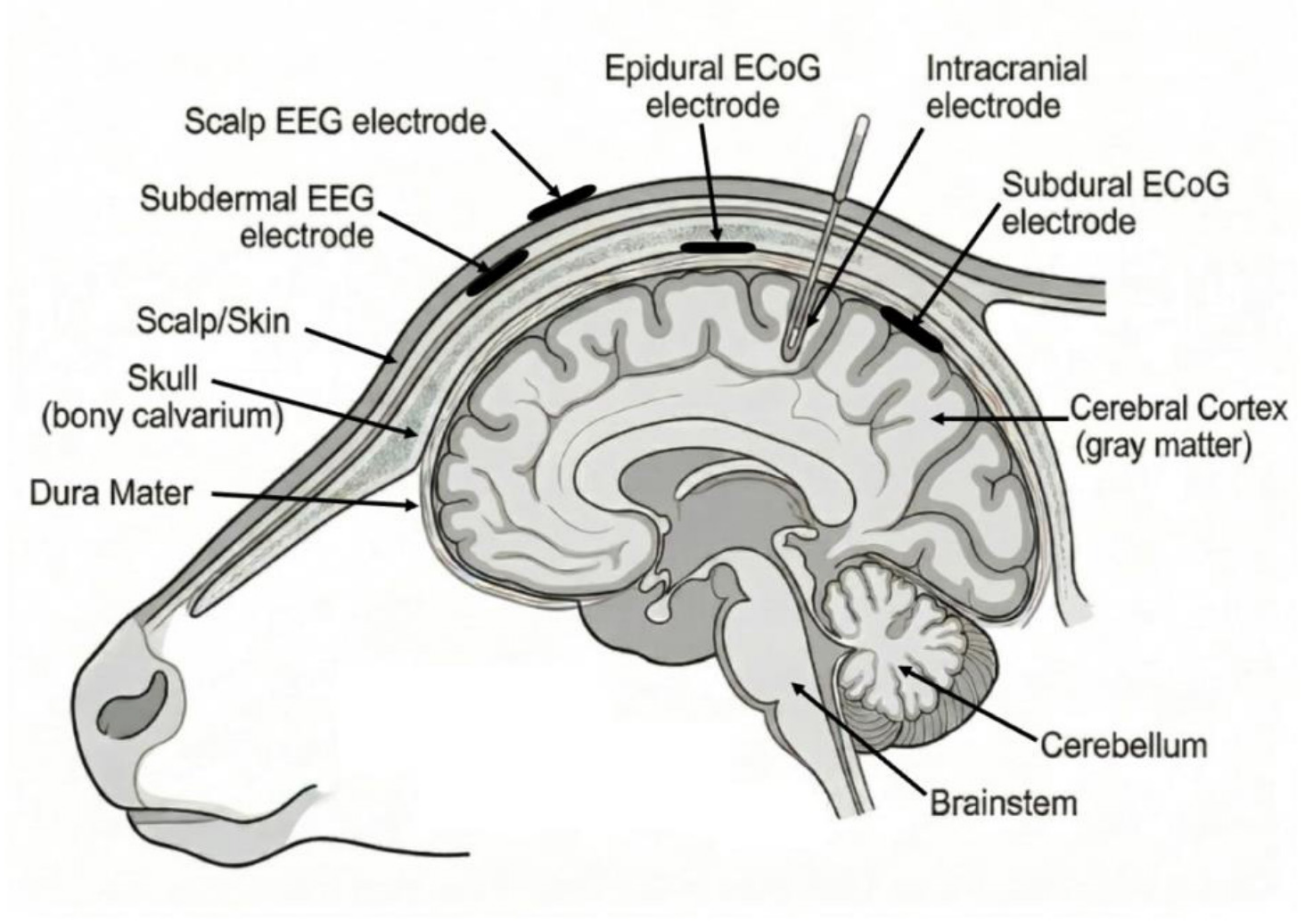
Schematic representation of EEG and ECoG electrode placement relative to the bovine skull and cerebral cortex.

EEG electrodes can be classified into several categories based on their anatomical position relative to the skull and brain tissue. Placement of the electrodes greatly affects the integrity of the recording (Figure 1).

- Non-invasive surface recording: Electrodes placed on the scalp surface.
- Minimally invasive subdermal recording: Electrodes positioned beneath the scalp but above the skull.
- Invasive electrocorticographic recording (ECoG): Electrodes surgically placed through the skull in various positions relative to the brain tissue: Electrodes may be surgically placed above the dura (epidural), below the dura (subdural), or within the cortex itself (intracranial).

### Scalp/Subdermal EEG recording

Scalp EEG is the most widely used modality of EEG testing in human clinical practice, given the ease of use. However, because scalp EEG electrodes are pasted or glued on to the head, they tend to slip, especially with movement, resulting in artifacts which obscure the recordings. More importantly, the skull presents a source of technical limitations for EEG recording, including variability related to skull thickness and a significant limitation on spatial resolution (45). However, advances in modern EEG methodology, including improved analog-to-digital conversion, signal filtering and electrode design have mitigated some of the historical limitations associated with subdermal and scalp recordings relative to ECoG.

Electrodes may also be placed below the scalp, above the skull, known as subdermal (or subscalp) electrodes. The use of subdermal electrodes reduces (but does not eliminate) the risk of movement artifact but cannot overcome the skull limitation.

### Electrocorticographic recording

ECoG utilizes electrodes that are placed through the skull and lie in or on the cerebral cortex itself. This technique provides the distinct advantage of significantly reducing movement artifact, resulting in a high signal to noise ratio. This technical benefit of ECoG makes ECoG especially useful for animal research. Being able to record high-quality data without movement artifacts is crucial in studies where behavioral restraint might be limited or challenging. As a result, many animal studies discussed below rely on ECoG to provide precise neurophysiological information.

### EEG vs. ECoG

**Figure 2:**
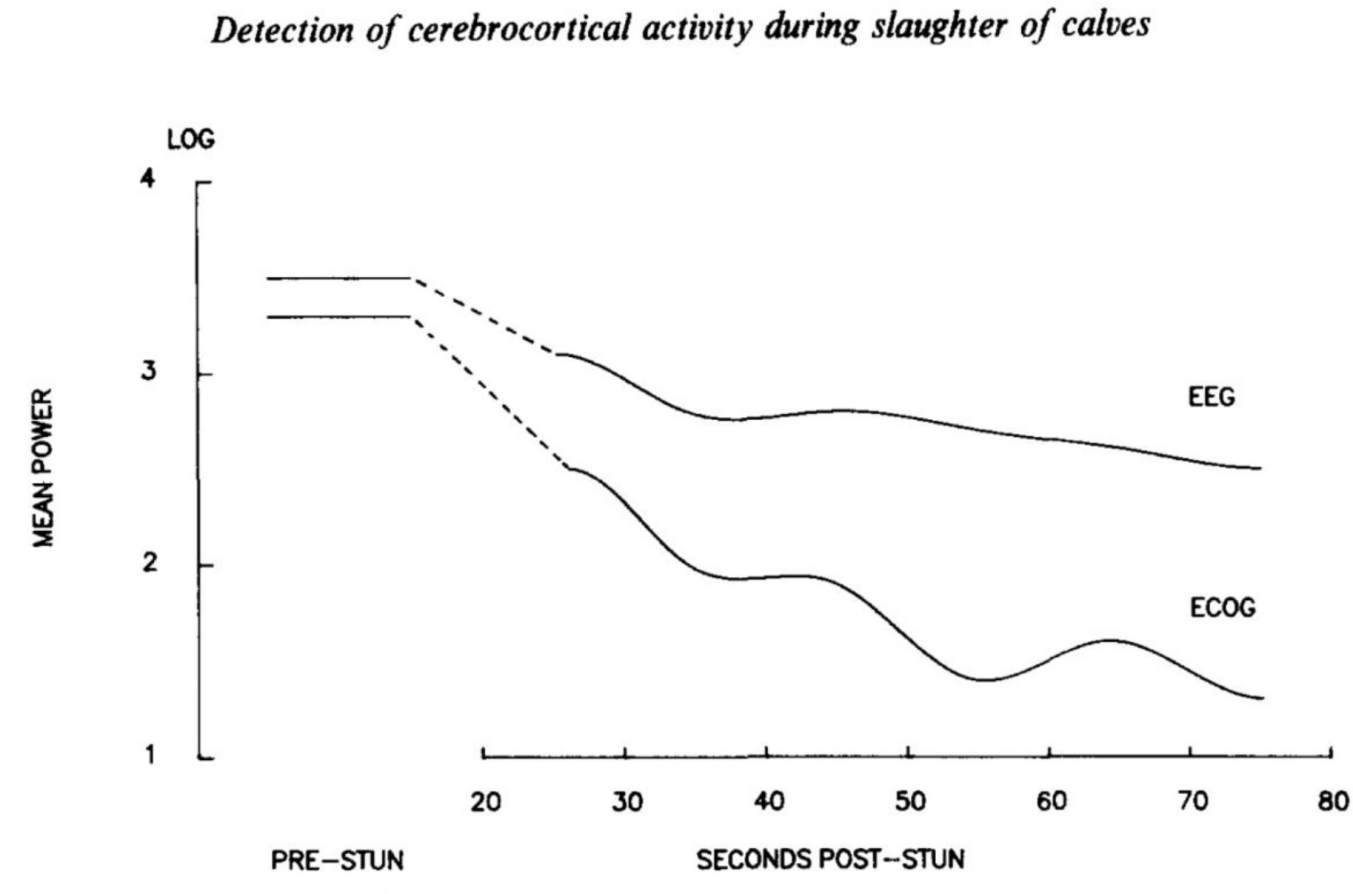
Percentage change in total power (P_tot_; a measure of overall cortical activity) of the EEG vs. ECoG. The slower rate of decline in the power content of the EEG as compared to ECoG will overestimate time to loss of cortical activity. [Reproduced with permission, 45].

Bager et al. (46) offered direct empirical evidence supporting the superiority of ECoG over surface EEG in evaluating animal consciousness. The investigators examined EEG and ECoG after neck cutting in halothane-anesthetized calves (46). As shown in Figure 2, comparison of the percentage change in total power (P_tot_), a measure of overall cortical activity, reveals clear differences between EEG and ECoG. Notably, ECoG provides a more sensitive and reliable assessment of the timing of cortical activity loss following neck cutting, indicating its superiority over EEG for this purpose. The slower decrease in EEG power during slaughter suggests that EEG-based assessments may overestimate the duration of cortical activity compared to ECoG, a finding supported by known differences in signal fidelity, spatial resolution, and susceptibility to artifact between scalp EEG and direct cortical recordings. These limitations of EEG are well established in the broader electrophysiology literature (47–49). Human studies also confirm the superiority of the signal obtained by ECoG over scalp electrodes (50).

### EEG interpretation

Visual inspection is the most common method of EEG interpretation, and the method utilized by the majority of the literature reviewed here. Interpretation is done by trained electroencephalographers, with reports generated describing the normal and abnormal patterns present in the recording, along with the significance of the findings. Typical EEG review occurs at the equivalent of 5–10 microvolts per millimeter. Evaluation for electrocerebral silence is suggested at 2 microvolts per division. At this level of amplification, many artifacts, from the heart, pulses and electrode or machine artifacts can imitate cortical EEG activity. Therefore, absence or proper recognition of these artifacts is necessary to call an EEG flat.

In spectral analysis, the frequencies that make up the EEG signal are quantified as the “power” of each component. A commonly used complex algorithm for this analysis is the Fast Fourier Transform (FFT), which portrays the EEG in terms of how much activity is present in different frequency bands (45). A full explanation of FFT is beyond the scope of this report but, simply put, applying an FFT algorithm to EEG recording computes the percentage of fast vs. slow frequencies in epochs of EEG. This method is utilized in a number of the papers reviewed here.

### Evoked potentials

EP are electrophysiologic signals representing time-locked cortical or subcortical responses to controlled sensory stimulation, such as somatosensory, auditory, or visual inputs. Typically, the sensory stimulus is delivered hundreds to thousands of times, with signal averaging of the results performed to eliminate the noise and preserve the time locked response (51). Unlike spontaneous EEG activity, EP assess the functional integrity of specific sensory pathways and their cortical projections rather than global cortical state. As such, EP indicate the presence of stimulus-driven and not spontaneous neural activity.

#### Measuring unconsciousness with neurophysiologic techniques

Consciousness spans states ranging from wakefulness to unconsciousness and ultimately to brain death. Electrophysiologically, LOC is marked by the onset of generalized high amplitude, low frequency (HALF) activity, reflecting global cortical dysfunction, whereas later findings such as isoelectric EEG represent complete cortical inactivity. Thus, LOC precedes these later endpoints and defines the point at which perception, including pain, is no longer possible.

### EEG frequency

Changes in sensorium occur along a spectrum between full awareness and unconsciousness, and efforts to characterize the EEG features of these states continue to evolve with the development of increasingly sophisticated techniques. Although EEG cannot precisely differentiate each of these states, EEG can reliably identify an awake state, an unconscious state and a state of brain death (52). While a normal awake EEG spans a broad frequency spectrum including alpha and beta rhythms (53, 54), a shift toward dominance of slow-wave (delta) activity has long been associated with reduced levels of consciousness. In fact, the association between slow waves/delta waves and an absence of consciousness has been known since at least the 1950′s (55). In their seminal work, “The Diagnosis of Stupor and Coma,” initially published in 1966, Plum and Posner emphasized that HALF patterns occur regularly in patients with depressed consciousness (56). This is repeated in most textbooks of EEG (57). Indeed, this was the principle driving the early animal slaughter studies many of which were performed in the 1960s and utilized HALF as a marker for unconsciousness in animals. More recent studies have utilized newer technologies including the Fourier analyses mentioned above, and even in these studies, an EEG dominated by delta waves continues to be considered the marker for LOC.

In humans, the earliest moments of unconsciousness typically rely on evidence of a behavioral change. Many studies aimed at identifying the precise moment of LOC in humans are found within the anesthesia literature. In one study for example, Gugino et al. (58) recruited healthy volunteers to undergo anesthesia accompanied by quantitative EEG analysis, while measuring levels of response to stimulation. While light sedation was associated with changes in other EEG frequencies, deep sedation was associated with slow wave delta and theta activity. A recent review of delta waves distinguishes between high amplitude delta oscillations which are associated with unconsciousness, and other forms of delta waves, typically lower amplitude ones, which are associated with other conditions (41). Although not directly comparable to EEG changes observed during acute cerebral hypoperfusion or hypoxia as in non-stun slaughter, the electrophysiological features associated with unconsciousness are broadly consistent across mechanisms and remain relevant.

### EEG Amplitude vs. frequency

Some studies rely on the amplitude of the EEG measurements rather than the frequency. The amplitude of activity recorded from awake EEG in an adult is typically reported as low (<20 uV), average (20–50 uV) or high (>50 uV) but these numbers are only relevant in reference to the background amplitude (59). Amplitude will also vary depending on age (being higher in children), montage and electrode placement, and impedance. Therefore, utilizing amplitude of EEG waves to identify LOC has significant limitations. Furthermore, it is known that 10% of normal human adults will, at baseline, have very low amplitude EEG recordings (60). In fact, experiments by Penfield and Jasper who performed intracranial EEG suggest an amplitude ratio of 10 to 1 when comparing direct cortical to surface EEG (60). Thus, EEG frequency appears to be more reliable than amplitude when assessing electrophysiological evidence of LOC, particularly with scalp EEG measurements.

### HALF vs. isoelectric EEG

An isoelectric EEG, or “flat line” EEG, reflects the absence of detectable cerebral electrical activity and is consistent with, though not itself diagnostic of, brain death in humans (61). In the context of non-stun slaughter in cattle, an isoelectric EEG clearly indicates that the animal is unconscious; however, it does not allow a determination of the onset of unconsciousness which necessarily occurs prior to brain death. Accordingly, in studies reporting time to isoelectric EEG, it is reasonable to infer that LOC occurred before the recorded time point (2, 62, 63). However, it is impossible to determine from studies that report only time to isoelectric EEG when unconsciousness began.

### Evoked potentials (EP)

As discussed above, EPs measure stimulus-driven neural activity and do not measure integrated cortical processing or conscious experience. EPs can provide information about cerebral cortical activity, the functional integrity of sensory pathways and cortical structures, and may help characterize levels of brain dysfunction that could support or preclude consciousness. However, EPs are commonly preserved during deep surgical anesthesia and are routinely used in intraoperative neurophysiological monitoring to assess neural pathway integrity in patients who are unequivocally unconscious (64, 65). Consequently, although EPs provide valuable information about neural conduction and pathway integrity, their utility for determining onset of LOC is limited, in much the same way as isoelectric EEG. Specifically, the loss of EPs implies that LOC must have occurred at some point prior, but does not indicate when LOC occurred (2).

## Methods

**Figure 3:**
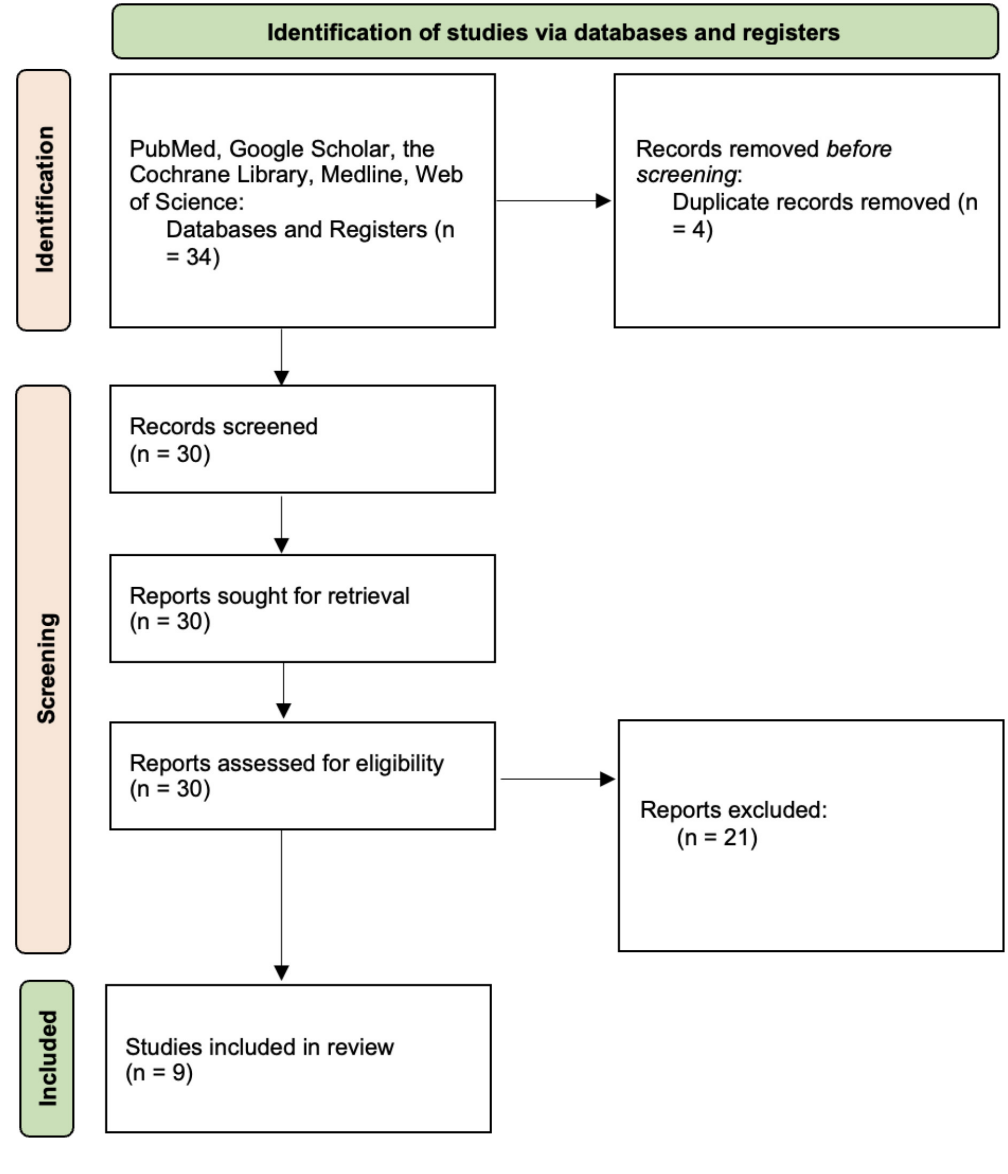
PRISMA flow diagram.

This systematic review was conducted in accordance with PRISMA guidance to identify and synthesize neurophysiologic studies evaluating LOC in cattle following slaughter with and without pre-slaughter stunning. A comprehensive literature search was performed in PubMed/MEDLINE, Web of Science, the Cochrane Library, and Google Scholar from database inception through December 2025. Search terms combined concepts related to bovine species, slaughter methods (including religious slaughter, shechita and halal), and electrophysiologic assessment of consciousness (EEG, ECoG, and EPs), using database-specific syntax and Boolean operators. Additional records were identified through manual screening of reference lists and targeted searches to capture foundational and historical studies not consistently indexed in electronic databases. A detailed description of the database specific search strings and supplemental identification methods is provided in Supplementary Appendix A. Eligible studies were original experimental investigations in cattle employing electrophysiologic techniques to assess consciousness during or following slaughter with and without pre-slaughter stunning. Review articles, studies relying solely on behavioral or reflex-based indicators, and studies involving non-bovine species were excluded. Titles and abstracts were screened for relevance, followed by full-text review. A total of nine studies met predefined inclusion criteria and were included in the qualitative synthesis. The study selection process is summarized in a PRISMA flow diagram (Figure 3). Due to heterogeneity in study design, electrophysiologic modality, and outcome definitions, results were synthesized qualitatively. Risk of bias was independently assessed at the study level using the ROBINS-I tool, with judgments made across standard bias domains and summarized narratively. The detailed ROBINS-I assessment is provided in Supplementary Appendix B. As previously mentioned, (See Section *Measuring unconsciousness with neurophysiologic techniques*) and for the purpose of this review, LOC was defined electrophysiologically as the onset of generalized HALF cortical activity, consistent with global cortical dysfunction. When studies did not explicitly report HALF activity, equivalent electrophysiologic markers of cortical suppression were interpreted in the context of the modality used, with recognition that endpoints such as isoelectric EEG or loss of evoked potentials represent later stages that necessarily follow LOC.

## Results

### ROBINS-I

Overall risk of bias across included studies was judged to be moderate, reflecting heterogeneity in study design, historical methodological limitations, and unavoidable confounding inherent to physiological experimentation. Importantly, studies at lowest risk of bias, particularly those employing invasive cortical recordings, demonstrated consistent and convergent evidence of rapid LOC following slaughter without stunning.

### EEG studies in non-stun slaughter of bovine species

Scalp and subdermal (or sub-scalp) EEG: Newhook, Blackmore and Grandin studied subscalp EEG and reported times to LOC in the bovines based on amplitudes below 10 uV or above 35 uV as inconsistent with consciousness (66, 67). Time to the appearance of these amplitudes had a wide range, from 28–168 s. These papers also reported a range of time to isoelectric EEG with a similarly wide range of 132–336 s. As discussed above, a significant limitation of these studies is the utilization of scalp EEG. The paper itself highlighted that movement artifact “obscured the EEG for 5–20 s” after the vessels were cut. The use of amplitude rather than frequency constitutes another limitation to these analyses.

Gibson et al. investigated cortical responses to slaughter by ventral-neck incision without prior stunning in minimally halothane-anesthetized calves by recording EEG activity before and after incision and analyzing spectral indices to assess whether the procedure constituted a noxious stimulus (4). Although the study was not designed to measure the onset of unconsciousness, the authors reported the emergence of HALF wave activity at a mean of 76 s after incision with a standard deviation of 26 s.

Lambooij et al. (68) utilized scalp electrodes to examine LOC in veal calves undergoing non-stun slaughter with neck cutting and captive bolt stunning. The EEG results were analyzed using the FFT technique to perform a correlation dimension analysis (CD) in which a drop of the CD score indicated more slow wave activity. They reported a lag of 80 s on average from a faster CD largely represented by beta and some alpha activity until a CD consistent with unconsciousness was achieved following slaughter without stunning. This study is subject to several limitations. In human EEG, beta activity is frequently present in non-awake states and in fact beta often increases at the onset of during drowsiness (69), raising the question of utilizing the high CD score to evaluate awake state. More importantly, muscle artifact as recorded by scalp electrodes during movement, will approximate beta activity even if the animal is losing consciousness.

Verhoeven et al. (70) evaluated the validity of commonly used clinical reflexes as indicators of unconsciousness in calves subjected to different stunning and non-stunned slaughter methods by comparing reflex loss with EEG-defined LOC. Although the primary objective of the study was not to determine time to LOC, calves undergoing non-stunned slaughter in either an upright position (*n* = 7) or an inverted position (*n* = 25) were continuously monitored with EEG, and LOC was defined as the transition to EEG activity dominated by HALF waves. Using this electrophysiologic criterion, LOC occurred at a mean of 109 ± 32 s after neck cut in upright non-stunned calves and 49 ± 25 s in inverted non-stunned calves.

### Studies utilizing surgically implanted electrodes (ECoG)

#### Epidural electrodes

Nangeroni and Kennett utilized epidural electrodes to record EEG to study the onset of unconsciousness in five calves following non-stun slaughter (71). The initial appearance of slow-wave activity, indicative of unconsciousness and defined by EEG frequencies of 2–10 Hz [referred to as cycles per second (cps)], occurred between 4.4 and 6.9 s following the incision. The isoelectric point on EEG was seen between 18.8–24.9 s in 4/5 calves and at 139.2 s in the remaining calf.

Schulze et al. similarly utilized epidural EEG electrodes to investigate the time to LOC in calves after slaughter without stunning (72). The paper does not describe the early EEG changes in the slaughter without stunning group, only commenting that the EEG immediately after the cut was unchanged. The authors comment that *loss of reactivity* occurred within 10 s of slaughter without stunning. The authors do not specify whether loss of reactivity applies to EEG or clinical reactivity of the animal. They do provide data for the isoelectric point, signifying brain death, which occurred by 23 s indicating that LOC had occurred earlier. The data for calves slaughtered with captive bolt stunning showed early delta activity consistent with immediate brain trauma, and a time of 28 s to isoelectric point in 4 calves.

#### Intracranial electrodes

Daly, Kallweit, and Ellendorf utilized ECoG electrodes to measure ECoG in equal numbers of adult cattle who underwent slaughter after captive bolt stunning and cattle that underwent slaughter without prior stunning (2). The measures examined included time to appearance of HALF activity, time to isoelectric point, and time to loss of EPs. In calves undergoing slaughter without stunning, mean time to HALF was 7.5 ± 2 s, with a range of 5–13 s. The mean HALF times for stunned animals were 10 ± 5, with a range of 4–17 s and the mean time to isoelectric point was 69.5 ± 1.5 s (range of 67–71 s) for the captive-bolt stunning group and 75 ± 48 s (range of 19–113 s) for the slaughter without stunning group. The data is also similar to what was observed by Schulze et al. (72). Somatosensory and visual evoked potentials were abolished immediately in the stunned group and persisted for 32–126 s and 20–102 s, respectively, in the non-stun slaughter group. Notably, the authors report that captive bolt stunning generated such extreme force that the surgically placed EEG electrodes were broken in 25% of stunned calves.

Kallweit, Ellendorf, Daly and Smidt (42) utilized ECoG to measure isoelectric point in cattle undergoing slaughter with captive bolt stunning (*N* = 6) and slaughter without stunning (*N* = 4). They reported both ECoG (experiment 1) and ECoG with EP data (experiment 2). Experiment 1 did not yield a difference in time to isoelectric point between slaughter with and without stunning. In Experiment 2, time intervals were measured from the neck incision to the onset of an isoelectric ECoG in animals slaughtered without stunning, and from both the time of stunning and the subsequent neck incision to isoelectric ECoG in the captive bolt–stunning group. In the slaughter without stunning group, isoelectric ECoG occurred at a mean of 10.8 s following the neck incision. In the captive bolt–stunning group, isoelectric activity occurred at a mean of 22.5 s after stunning and 5.8 s after the subsequent neck incision. Although isoelectric ECoG was reached later in the stunning group, the differences in mean values between the groups were not statistically significant (ANOVA, *p* < 0.05) for either comparison.

Similar to Daly et al. (2), EPs were completely absent following captive bolt stunning, while somatosensory and visual EPs persisted for 77 s and 55 s, respectively, after slaughter without stunning.

Bager et al. (43) utilized intracranial electrodes to record ECoG in 3 groups of calves. Group 1 underwent electrical stunning, followed by a period when the animals were allowed to recover consciousness. This was then followed by repeat electrical stunning and ventral neck incision. Group 2 underwent electrical stunning without recovery followed by ventral neck incision and Group 3 underwent slaughter by ventral neck incision without stunning. The aim was to determine time to the appearance of HALF waves and to the isoelectric point. The authors report that in Group 3 pre-slaughter EEG showed high frequency activity (as expected). Following cutting, the high frequency activity “reduced considerably in amplitude and was close to isoelectric values in approximately 15 s. The low frequency activity was maintained longer and in 3 cases there appeared to be a burst of activity at 10 s.” The timing to isoelectric point for the 3 groups was 48 ± 5 s for Group 1, 54 ± 9 s for Group 2, and 44 ± 3 s in Group 3.

The study included an analysis to determine FFT power in the groups. The authors considered frequencies between 8–30 Hz to be “high frequency” cortical activity consistent with sensibility (consciousness) and frequencies below 8 Hz to represent insensibility (unconsciousness). The animals in Group 3 are described as exhibiting a “dramatic drop (in frequency) within a few seconds of slaughter (time 0) indicating that sensibility (consciousness) was rapidly lost.” In half of the animals, the low frequency activity also fell quickly toward isoelectric point, and in the other half there was a more prolonged period of unconsciousness until isoelectric point was reached.

## Discussion

A central challenge in the humane slaughter debate is that consciousness in animals cannot be measured directly. In the absence of a definitive biomarker, investigators rely on surrogate measures, including electrophysiologic recordings and clinical or behavioral observations, each of which carries inherent limitations. Among these approaches, electrophysiology provides the most objective and temporally precise assessment of cortical function. However, not all electrophysiologic modalities are equally informative for evaluating LOC. To our knowledge, this review is the first to provide a foundational overview of EEG, ECoG, and EP specifically tailored to the context of cattle slaughter, addressing a critical gap in the existing literature. The absence of this interpretive framework may help explain discrepancies in earlier reports, including the wide range and sometimes inconsistent estimates of time to LOC following slaughter without stunning (14, 30, 32, 34). In interpreting these findings, it is important to emphasize the specific scope of the evidence synthesized in this review. The analysis addresses one clearly defined component of animal welfare at slaughter, namely the time to LOC following the non-stun slaughter incision as measured by electrophysiological testing. This is directly relevant to regulatory and welfare evaluations concerned with the rapidity of insensibility. As outlined earlier, scalp EEG and EP have important interpretive limitations in the slaughter setting and are poorly suited for determining the timing of LOC. In contrast, direct cortical recordings using ECoG provide the most reliable and temporally precise assessment of cortical function and therefore form the strongest electrophysiologic basis for evaluating LOC in these studies. When carefully evaluated, the available evidence from ECoG studies indicates that the onset of HALF activity, interpreted in this context as a marker of loss of cortical function consistent with unconsciousness, most often occurs within approximately 4–13 s in higher-fidelity recordings. It is important to contextualize these findings within the broader debate regarding animal welfare in slaughter without stunning. While the electrophysiologic data summarized here support rapid LOC, this does not imply that all aspects of the slaughter process are free from welfare concerns. Factors such as pre-slaughter handling, restraint methods, and technical aspects of the incision, including blade sharpness and cut placement merit comprehensive welfare evaluation but are outside the scope of this review. It is also important to distinguish between commonly reported electrophysiologic endpoints and those that are physiologically most informative for determining the timing of LOC. While isoelectric EEG has frequently been reported in the slaughter literature, it reflects complete cessation of cortical activity and therefore represents a late endpoint that necessarily follows earlier LOC. In contrast, the emergence of generalized HALF activity reflects acute global cortical dysfunction and is more temporally aligned with the onset of unconsciousness. However, these findings should be interpreted in the context of several methodological limitations across the included studies. Sample sizes were generally small, and substantial variability in reported times to LOC was observed, particularly in studies using scalp EEG. This variability likely reflects differences in electrophysiologic modality, electrode placement, animal condition (e.g., anesthesia, restraint), and procedural factors, all of which can influence the temporal detection of cortical activity. A particularly instructive example of the methodological limitations inherent to EEG, is provided by the study of Verhoeven et al. (70). In that study the concept of an initial “transitional zone” of LOC, marked by the presence of non-dominant HALF waves is introduced. Although transitional EEG states are recognized and discussed in both animal and human studies, this transitional-phase interpretation is not present in the electrophysiology literature on cattle in the context of slaughter by ventral neck incision. Verhoeven et al. (70) cite McKeegan et al. (73) as evidence for a “transitional zone”; however, that study examined behavioral and physiological responses of poultry exposed to gas-filled high-expansion foam, a different species and a stunning method known to induce unconsciousness gradually, and therefore one in which a transitional phase would be expected. Furthermore, in the Verhoeven et al. (70) dataset, loss of cortical-mediated reflexes, as inferred from the disappearance of the threat and withdrawal reflexes, frequently occurred before the EEG-defined transitional zone proposed by the authors. Likewise, loss of brainstem reflexes such as corneal and palpebral responses, which are often interpreted as markers of brain death, was sometimes observed before the EEG-defined LOC point. These sequences are difficult to reconcile with established neurophysiological principles and are highly suggestive that scalp EEG lags behind clinical and physiological indicators in detecting the true time to onset of LOC. This delay may in part reflect the placement of surface electrodes, as well as inherent limitations of EEG itself as discussed above, including limited spatial resolution, susceptibility to motion artifact, and reduced temporal precision relative to ECoG.

In a recent systematic review, Hascalovici (44) presented neurophysiologic and behavioral studies of cattle slaughter and rated quality of the studies using a custom hierarchy of evidence scale. Our review arrived at similar conclusions, namely that the highest quality evidence deduced from ECoG studies suggests that LOC in cattle undergoing slaughter without stunning occurs in the range of 4.4 s to 13 s, with the mean time ranging from 7.5 s to 10.8 s (2, 29, 42, 43, 71) (Table 1).

**Table 1:** Summary of neurophysiologic studies of loss of consciousness following non-stun slaughter, adapted from Hascalovici et al. (29) (Reproduced with permission).

	Author	Animal	Slaughter	Sample (N)	Endpoints	Results (seconds)
EEG	Nangeroni and Kennett (71)	Calves	Shechita	N=5	Delta waves:	4.4-6.9
					Isoelectric point:	20 (N=4) 120 (N=1)
	Schulze et al. (72)	Calves	Religious (unspecified)	N=10	Loss of reaction:	≤ 10
					Isoelectric point:	≤ 23
	Newhook and Blackmore (66)	Calves	Non-stun, non-religious	N=8	Low voltage fast activity < 10uV or >35uV:	34-85
					Isoelectric point:	132-336
	Gibson et al. (4)		Non-stun, non-religious with halothane anesthesia	N=14	HALF	76 seconds (SD 26)
	Lambooij et al. (68)	Calves	Non-stun, non-religious	N=10	Delta (0-4 Hz) & theta (4-7 Hz) waves:	80
	Verhoeven et al. (70)	Calves		Upright: N=7 Inverted: N=25	HALF wave dominant EEG:	Upright: 109 ± 32 Inverted: 49 ± 25
ECoG	Gregory and Wotton (74)	Calves	Non-stun, non-religious with halothane anesthesia	N=8	Loss of EP:	(mean ± SD) 17 ± 4
					Isoelectric point:	23 ± 11
	Daly et al. (2)	Adult Cattle	Shechita	N=8	Loss of somatosensory EP:	(mean ± SD) 77 ± 32
					Loss of visual EP:	55 ± 32
					HALF:	7.5 ± 2
					Isoelectric point:	75 ± 48
	Kallweit et al. (42)	Adult Cattle	Shechita	N=4	Loss of ECoG activity:	10.8
	Bager et al. (43)	Calves	Non-stun, non-religious	N=6	HALF:	< 10
					Isoelectric point:	44 ± 3

[Refers to citation in Discussion paragraph 3].

This study has several limitations that merit acknowledgment. The number of eligible electrophysiologic studies is relatively small, reflecting the limited size of the available literature. In addition, heterogeneity across studies, including differences in animal age, restraint conditions, anesthesia use, slaughter environments, and electrophysiologic techniques, precluded quantitative meta-analysis and necessitated a qualitative synthesis. Electrophysiologic techniques, particularly invasive methods such as ECoG, today face the challenge of more robust ethical research guidelines. This likely contributes to the relative scarcity of ECoG-based studies, despite their superior signal fidelity and temporal precision for assessing cortical function. This underscores the value of the existing ECoG literature, as such studies are unlikely to be repeated at scale in the future, thereby elevating the relevance of past studies in informing current understanding. Broader welfare considerations at slaughter include factors such as pre-slaughter handling, restraint, cut quality, and operator proficiency, all of which may influence the animal's overall experience. These factors are important components of comprehensive welfare assessment but are outside the scope of the present study. This review contributes to the literature by providing a detailed neurophysiologic framework and systematic synthesis relevant to evaluating time to LOC during cattle slaughter, an issue of clear importance to animal welfare discussions. By integrating a foundational overview of electrophysiologic methodology with best available evidence, the review helps contextualize prior reports that have described wide and sometimes prolonged estimates of time to LOC following slaughter without stunning. When considered in light of invasive cortical recordings, the best available electrophysiologic data is inconsistent with a progressive LOC over extended periods; rather, it suggests that LOC occurs rapidly and, in some studies, within a timeframe comparable to, or in at last one study even shorter than, that observed following stunning (2).

## Conclusions

This review supports the conclusion that slaughter without stunning results in rapid loss of consciousness. This data aligns with core animal welfare goals of minimizing suffering and may inform the ethical and regulatory evaluations of ritual slaughter. As the field of neurophysiology develops even more sophisticated techniques, future studies may examine these issues with even greater clarity.

## Data Availability

The original contributions presented in the study are included in the article/Supplementary material, further inquiries can be directed to the corresponding author.
