# Supplementary material for: Electrophysiologic evidence of loss of consciousness in cattle during slaughter with and without stunning: a systematic review and methodological overview
Source: Front Vet Sci. 2026 May 18;13:1809389. doi: 10.3389/fvets.2026.1809389 (PMC13224944; doi:10.3389/fvets.2026.1809389)
Supplement: Supplementary file 1 [file Table_1.docx]

**Supplementary Appendix A**
 **Detailed Literature Search Strategy**

**Databases Searched**: PubMed / MEDLINE; Web of Science Core Collection; Cochrane Library; Google Scholar

**Search Period**
From database inception through December 31, 2025

**PubMed / MEDLINE Search Strategy**

The following search string was used in PubMed / MEDLINE:

(bovine OR cattle OR calf) AND
(slaughter OR "religious slaughter" OR shechita OR ritual OR exsanguination OR "neck cutting" OR stunning) AND
(electroencephalography OR EEG OR electrocorticography OR ECoG OR "evoked potentials") AND
("loss of consciousness" OR unconsciousness OR insensibility OR "cortical activity")

**Web of Science Core Collection Search Strategy**

The following topic-based search string was used in the Web of Science Core Collection:

TS = (bovine OR cattle OR calf) AND
TS = (slaughter OR "religious slaughter" OR shechita OR ritual OR exsanguination OR "neck cutting" OR stunning) AND
TS = (EEG OR electroencephalography OR ECoG OR electrocorticography OR "evoked potentials") AND
TS = ("loss of consciousness" OR unconsciousness OR insensibility OR "cortical function")

**Cochrane Library Search Strategy**

The following search string was used in the Cochrane Library:

(bovine OR cattle OR calf) AND
(slaughter OR shechita OR exsanguination OR "neck cutting" OR stunning) AND
(electroencephalography OR EEG OR electrocorticography OR "evoked potentials")

**Google Scholar Search Strategy**

The following search terms were used in Google Scholar:

bovine OR cattle AND
slaughter OR shechita OR exsanguination AND
EEG OR ECoG OR electrocorticography OR "evoked potentials" AND
consciousness

**Additional Identification Methods**

To ensure comprehensive coverage, additional studies were identified through manual screening of reference lists from included articles and relevant review papers. Targeted searches were also performed to capture foundational and historical studies published prior to widespread electronic indexing, including early and mid-20th century literature. Non-English publications were considered when English abstracts or reliable translations were available.
