# Supplementary material for: Electrophysiologic evidence of loss of consciousness in cattle during slaughter with and without stunning: a systematic review and methodological overview
Source: Front Vet Sci. 2026 May 18;13:1809389. doi: 10.3389/fvets.2026.1809389 (PMC13224944; doi:10.3389/fvets.2026.1809389)
Supplement: Supplementary file 2 [file Table_2.docx]

**Supplementary Appendix B
Risk of Bias Assessment (ROBINS-I Framework)**

Risk of bias in the included studies was assessed using the ROBINS-I (Risk Of Bias In Non-randomized Studies of Interventions) framework, adapted for experimental, observational, and physiological studies examining slaughter and loss of consciousness in cattle. Each study was evaluated across the seven ROBINS-I bias domains. Given the historical nature and methodological heterogeneity of the literature, assessments were qualitative and domain based rather than numerical.

1. **Bias Due to Confounding**

*Moderate risk of bias*

Several studies were subject to important confounders, including the use of anesthesia, carotid clamping rather than carotid sectioning, variations in animal positioning such as hoisting, and differences in the timing of post-incision measurements. These factors may influence cerebrovascular pressure, cerebral blood flow, and electrophysiologic signals. However, despite these confounders, the direction and timing of the primary physiological effects were consistent across studies, reducing the likelihood that confounding alone explains the observed findings.

1. **Bias in Selection of Participants into the Study**

*Low to moderate risk of bias*

All included studies examined cattle or calves. Animals were typically selected based on availability rather than random sampling, and allocation procedures were rarely described. Because outcomes consisted of objective physiological and electrophysiologic measurements rather than subjective assessments or between-group comparisons, selection bias is unlikely to have materially affected internal validity.

1. **Bias in Classification of Interventions**

*Low risk of bias*

Interventions, including religious slaughter methods, carotid sectioning, carotid clamping, vertebral artery manipulation, and stunning techniques, were generally well defined and clearly distinguishable. Misclassification of intervention status was unlikely, particularly in studies involving direct surgical manipulation or explicitly described slaughter protocols.

1. **Bias Due to Deviations from Intended Interventions**

*Moderate risk of bias*

Some studies deviated from conditions representative of commercial slaughter, including the use of anesthesia, experimental vascular clamping rather than sectioning, or laboratory-based settings. These deviations limit direct extrapolation to real-world slaughter conditions but do not undermine mechanistic conclusions regarding rapid cerebral pressure collapse, flow redistribution, and cortical hypoperfusion following interruption of cerebral circulation.

1. **Bias Due to Missing Data**

*Low risk of bias*

Most studies reported complete outcome data for the physiological and electrophysiologic variables measured. Attrition and missing data were uncommon and, when present, were not clearly associated with outcome direction or study conclusions.

1. **Bias in Measurement of Outcomes**

*Low to moderate risk of bias*

Studies using direct quantitative measures, including arterial pressure, blood flow, and electrophysiologic recordings, were judged at low risk of measurement bias. In contrast, studies relying on indirect or qualitative indicators, such as visual assessment of dye distribution or behavioral observation, were considered at moderate risk due to potential observer subjectivity, movement artifact, and lack of standardized thresholds. Blinding of outcome assessors was generally not reported.

1. **Bias in Selection of the Reported Result**

*Moderate risk of bias*

Because preregistered protocols were not available, selective reporting cannot be excluded. However, the inclusion of studies with variable findings, explicit discussion of methodological limitations, and results both supportive and non-supportive of rapid loss of consciousness reduces concern for systematic reporting bias favoring a single conclusion.

**Overall Risk of Bias Judgment**

Overall, the body of evidence was judged to have a **moderate risk of bias**. This reflects heterogeneity in study design, historical methodological limitations, and unavoidable confounding inherent to physiological experimentation. Importantly, no single study at critical risk of bias dominated the evidence base, and conclusions were supported by convergent findings across independent anatomical, hemodynamic, electrophysiological, and behavioral approaches, strengthening confidence in the overall interpretation.
